# Insights on Prevalence of Hereditary Aortic Thoracic Disease and Impact on Family Screening in Routine Clinical Cardiothoracic Surgery Care

**DOI:** 10.3390/genes17091013

**Published:** 2026-08-27

**Authors:** Simone Gasser, Michael Graber, Nikolaos Bonaros, Michael Grimm, Julia Dumfarth

**Affiliations:** 1 University Clinic for Cardiac Surgery, Medical University Innsbruck, 6020 Innsbruck, Austria; 2 Department of Cardiac and Thoracic Aortic Surgery, Medical University of Vienna, 1090 Vienna, Austria

**Keywords:** genetic aortopathy, aortic dissection, aortic aneurysm, genetic testing

## Abstract

**Objective:** Heritable thoracic aortic disease (HTAD) is an important cause of thoracic aortic aneurysm and dissection, yet remains underdiagnosed. We aimed to summarize the prevalence of syndromic and non-syndromic HTAD, describe variants of uncertain significance (VUS), and evaluate the impact of family screening in a contemporary cardiothoracic surgical cohort. **Methods:** Between 2018 and 2026, 163 patients underwent genetic testing for suspected HTAD at our institution. A total of 139 patients with completed genetic testing were included in the analysis. Indications for testing included root aneurysm or acute aortic dissection before the age of 60 years with or without positive family history, aneurysms involving multiple vascular territories, or syndromic features suggestive of a heritable aortopathy. **Results:** The mean age at testing was 45.8 ± 15.1 years and 33% of patients were female. Aortic dissection represented the initial manifestation of disease in 40% of patients. Positive family history was present in 60% of patients. Pathogenic variants were identified in 24%. Twenty-three patients were diagnosed with syndromic HTAD, most commonly Marfan syndrome, while 10 patients were found to have non-syndromic HTAD. Variants of uncertain significance were detected in 27 patients (19%), predominantly affecting *MYLK* and *MYH11*. Following diagnosis, 87 relatives underwent screening, resulting in 18 prophylactic aortic operations. **Conclusions:** Cardiac surgeons should be aware of the importance of genetic testing in patients with aortic disease. Beyond the individual patient, the broader goal is to identify at-risk family members and prevent acute aortic syndromes. Continued collection of genotype–phenotype data is essential to improve interpretation of variants of uncertain significance and optimize future patient care.

## 1. Introduction

Genetic testing is recommended in patients with thoracic aortic disease diagnosed before the age of 60 years, those with a positive family history of thoracic aortic disease, aneurysms in other arterial segments, or syndromic features suggestive of a heritable aortopathy [1]. Currently, more than 60 genes have been found to be potentially causative of thoracic aortic disease (TAD) [2]. According to ClinGen, the following 11 genes have a definitive or strong association with heritable thoracic aortic disease (HTAD): *ACTA2*, *COL3A1*, *FBN1*, *LOX*, *MYH11*, *MYLK*, *PRKG1*, *SMAD 3*, *TGFB2*, *TGFBR1* and *TGFBR1* [3,4]. However, current genetic sequencing identifies potential causative genes in only 20% of patients tested [3]. Chen et al. reported that a positive family history of aortic dissection (AD) in a first-degree relative was associated with a 6.56-fold increased risk of AD, after excluding patients with Marfan syndrome and bicuspid aortopathy [5]. The risk was highest among individuals with an affected sibling. The heritability of AD was estimated to be 57% in this study including close to 24,000 patients [5]. Familiar aneurysms were found to be more malignant than sporadic aneurysms by the Yale group already over 20 years ago, with an earlier presentation and faster yearly aortic growth [6]. The same working group reported that at least 50% of patients suffered from aortic dissection within a five-year window before or after the age at which an affected family member had experienced a dissection [7]. Regalado et al. illustrated the complexity of individualized aortic care by demonstrating differences in the risk of aortic events associated with specific mutations in seven rare non-syndromic genes. Their findings highlight the importance of genetic counselling following diagnosis and of interdisciplinary collaboration in the management of patients with heritable aortic disease [8]. Taken together, the available evidence highlights the importance of genetic testing in cardiac surgery and the key role of cardiac surgeons as the main treating physicians in identifying patients who may benefit from genetic testing. The aim of our report was to provide real-world insight into the prevalence of heritable thoracic aortic disease (HTAD), the impact of family history, and the role of family screening in contemporary aortic care, based on our single-centre experience.

## 2. Patients and Methods

### 2.1. Study Cohort

**Figure 1:**
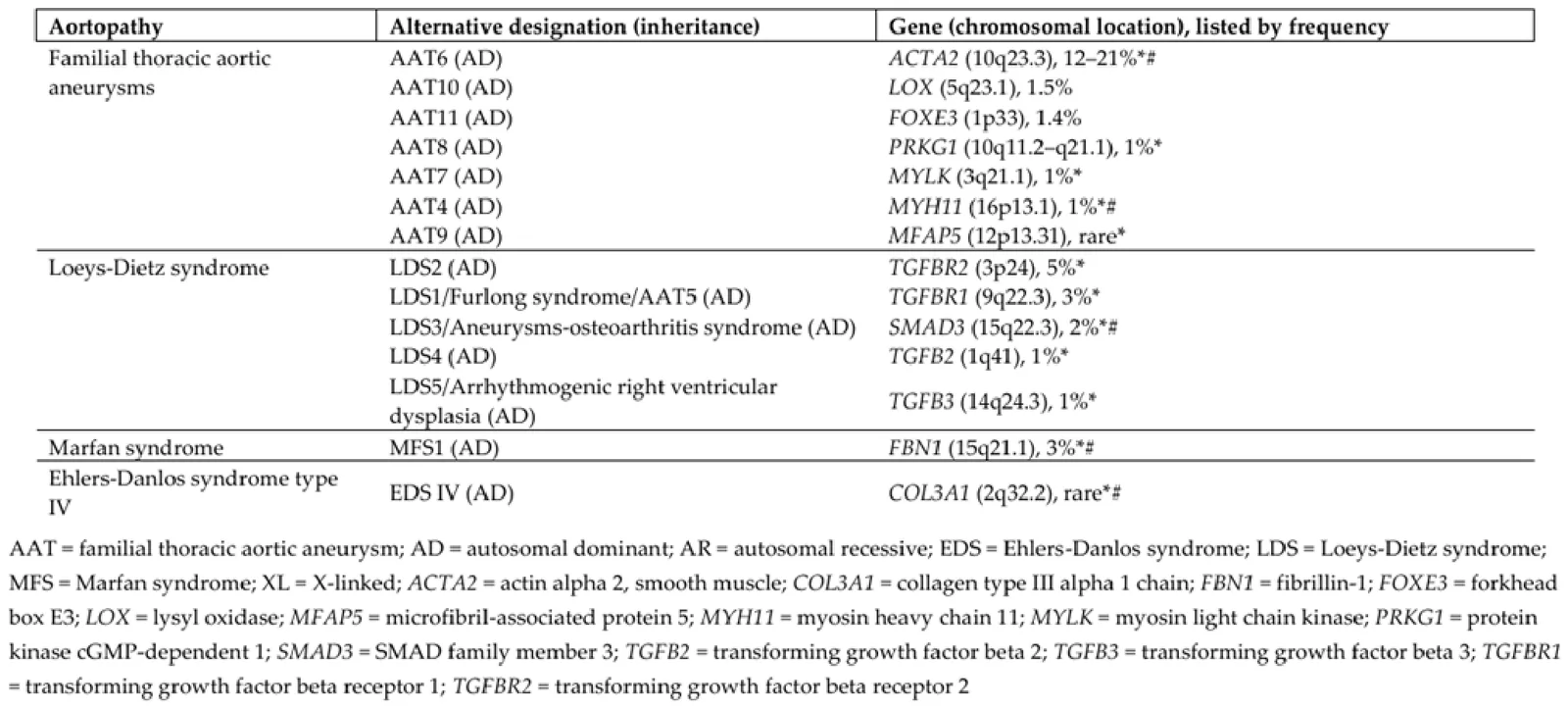
Our genetic testing panel used for the evaluation of hereditary thoracic aortic disease.

Between 2018 and 2026, 163 patients underwent genetic testing for hereditary thoracic aortic disease (HTAD) at our centre. Testing was performed by massive parallel sequencing of 14 genes with Nextera Rapid Capture (TruSight™ One; Illumina, Inc., San Diego, CA, USA) and NextSeq (Illumina, Inc., San Diego, CA, USA); for details on genes tested see Figure 1 [9]), and is covered by public national insurance. Variant classification was performed at the Center for Medical Genetics Innsbruck according to the American College of Medical Genetics and Genomics (ACMG) criteria, without application of ClinGen gene-specific specifications. Variants were classified at the time of genetic testing and were independently reviewed by three clinical geneticists. For variants of uncertain significance (VUS variant analysis was repeated five years after the initial analysiswhen recommended by the clinical geneticists, taking into account current evidence and databases, and the classification was updated where appropriate.

Testing was performed at our centre if patients suffered from acute aortic dissection or aortic root aneurysm with tricuspid valve at age < 60 years, a positive family history in a second- or first-degree family member of TAD or peripheral/intracranial aneurysm were evident, if arterial aneurysms were also present in other segments or aneurysm size progression was rapid and in those with aneurysm/dissection and syndromic features [1,10].

Family history was defined as followed:

(a) Proven: pathogenic/likely pathogenic variant in genetic testing or established syndromic diagnosis of a first-degree family member.
(b) Likely: at least two biologically related family members were affected by thoracic aortic aneurysm or at least one first-degree family member suffered from aortic dissection at a young age, suggesting familial/heritable disease, but no causative genetic variant had been identified.
(c) Possible: suggestive family history or clinical features raising suspicion of heritable disease, e.g., sudden unexplained death at young age of a first-degree family member or aneurysms of second-degree family members.

Non-syndromic HTAD (nsHTAD) was defined as thoracic aortic disease in patients carrying a pathogenic or likely pathogenic variant in an HTAD-associated gene, in the absence of clinical features fulfilling diagnostic criteria for a recognized syndromic aortopathy.

Information on family history was obtained when genetic testing was performed at our outpatient clinic or if missing, then via telephone interviews.

All patients diagnosed with HTAD were offered genetic counselling at the Institute of Human Genetics. Genetic counselling, cascade genetic testing, and aortic imaging was recommended for all first-degree relatives of individuals with confirmed HTAD. In addition, patients with a variant of uncertain significance (VUS) received genetic counselling, including a recommendation for aortic imaging of their first-degree relatives. Echocardiographic screening was also recommended for first-degree relatives of patients with a negative genetic test result but a positive family history and aortic events suggestive of hereditary disease [11]. Data on family screening after diagnosis was obtained via telephone interviews.

### 2.2. Statistical Analysis

IBM SPSS Statistics Version 24 (IBM Corp., Armonk, NY, USA) was used for statistical analysis. Continuous variables were tested for normal distribution using Kolmogorov–Smirnov test first and are either expressed as mean ± standard deviation or median and interquartile range. Categorical variables are shown as frequencies with corresponding percentages.

## 3. Results

**Figure 2:**
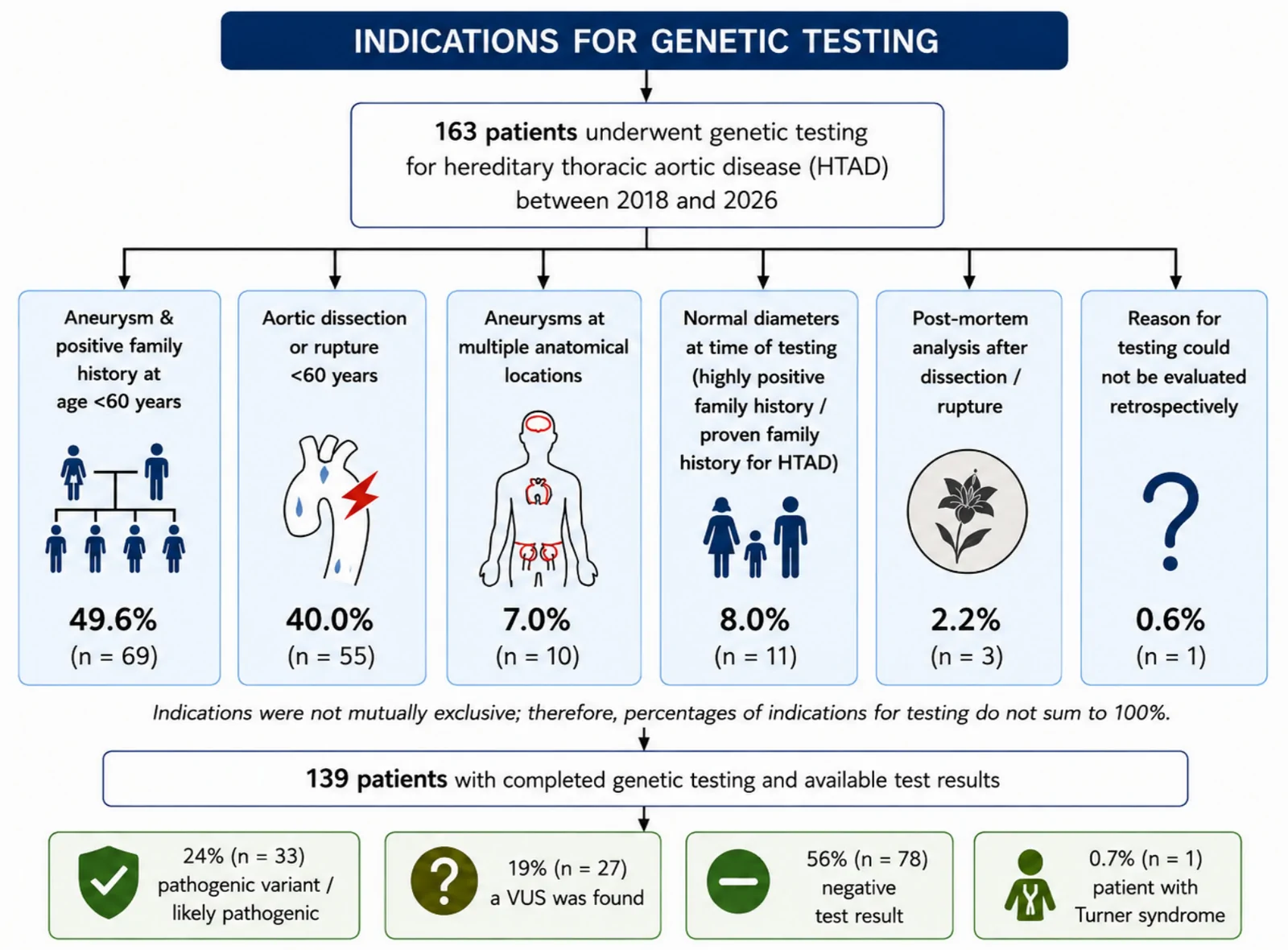
Flow chart for indication of genetic testing and summary of genetic findings.

A total of 139 patients with completed genetic testing and available test results were analyzed. Median age was 45.8 ± 15.1 years at timepoint of testing and 33% (n = 46) of patients were female. All included patients but one were of White ethnicity, predominantly of Central European origin (95%; n = 134) and five percent of patients (n = 5) were of Turkish origin. Twenty-four percent (n = 33) of patients were identified to carry a pathogenic variant and in 19% (n = 27) a VUS was found. One patient was diagnosed with Turner syndrome. For more details, see Figure 2 and Table 1.

### 3.1. Indications for Genetic Testing

Aneurysm (aortic root or ascending combined with positive family history) at an age < 60 years was the most prevalent reason for testing (49.6%; n = 69). Forty percent (n = 55) of patients were tested after surviving an aortic dissection or contained rupture. Seven percent (n = 10) presented with multiple aneurysms at different anatomical locations. In 2% of patients (n = 2), analysis was performed post-mortem after dissection/rupture. In one patient, reason for testing could not be evaluated retrospectively. Eight percent of patients (n = 11) had normal diameters at timepoint of testing and were screened due to highly positive family history/proven family history for HTAD. Indications were not mutually exclusive; therefore, percentages of indications for testing do not sum to 100%. For more details, see Table 2 and Figure 2.

Twelve percent (n = 16) had a proven family history. In 30.2% of patients (n = 42), family history was likely, and in 18% (n = 25) family history was possible. Fifty-three patients (38.1%) underwent genetic testing despite having no family history, based on clinical features suggestive of HTAD. In three patients, details on family history could not be evaluated. More details on family history are displayed in Table 2.

### 3.2. Syndromic Hereditary Thoracic Aortic Disease

Over the past 12 years, 23 patients with syndromic HTAD were identified at our cardiothoracic surgery department. Marfan syndrome was the most prevalent diagnosis (n = 11), followed by Loeys–Dietz syndrome (n = 8) and vascular Ehlers–Danlos syndrome (n = 4). For more details, see Table 1.

### 3.3. Non-Syndromic Hereditary Thoracic Aortic Disease

A diagnosis of non-syndromic HTAD was established in ten patients. Among patients with non-syndromic HTAD, pathogenic *MYH11* variants were identified in seven patients, while three patients carried a pathogenic *ACTA2* variant. Details are displayed in Table 1.

### 3.4. Variants of Unclear Significance

In our cohort, 19.4% of patients (n = 27) were found to carry a variant of still uncertain significance (VUS). Two patients were found to carry more than one variant of uncertain significance in different HTAD-associated genes; one patient presented two VUS of the same gene. Variants of uncertain significance were identified in *MYLK* (n = 11), *FBN1* (Marfan syndrome-associated gene; n = 6), *MYH11* (n = 6), *TGFBR1*/*TGFBR2* (Loeys–Dietz syndrome-associated genes; n = 3), *COL3A1* (vascular Ehlers–Danlos syndrome-associated gene; n = 2), MFAP5 (n = 1), and *LOX* (n = 1). A detailed overview of the clinical presentation and family history of patients with a variant of uncertain significance is provided in Table 3.

### 3.5. Family Screening, Preventive Aortic Surgery and Follow-Up Events

Following the recommendations outlined in the Methods section, a total of 87 first- and second-degree relatives of index patients carrying either a pathogenic/likely pathogenic variant or a variant of uncertain significance (VUS) underwent family screening by imaging and/or genetic cascade testing. Genetic cascade testing identified 32 relatives who carried the familial pathogenic/likely pathogenic variant detected in the respective index patient. In addition, 10 relatives were found to carry the familial VUS identified in the respective index patient. An additional 28 relatives of patients with negative test results underwent imaging-based screening. Of the relatives who underwent family screening, 18 subsequently underwent prophylactic aortic surgery based on the detection of clinically relevant aortic disease. Genetic findings alone did not constitute an indication for prophylactic surgery. Preventive aortic surgery was performed only in patients with documented aortic enlargement. Surgical decisions were based on aortic dimensions and current guideline recommendations, taking gene-specific surgical thresholds into account (1,2,10). Despite the diagnosis established previously in their affected relatives, five family members experienced disease-related adverse events, including three disease-related deaths and two aortic dissections. For more details, see Table 4.

## 4. Discussion

In this single-centre cohort of patients undergoing genetic testing for suspected hereditary thoracic aortic disease (HTAD), clinically actionable pathogenic or likely pathogenic variants were identified in a substantial proportion of patients, while an additional proportion carried variants of uncertain significance requiring careful interpretation and longitudinal clinical follow-up. This finding highlights the substantial contribution of heritable disease to thoracic aortic pathology encountered in cardiothoracic surgical practice. The fact that 40% of patients in our cohort presented with aortic dissection as the initial manifestation of their disease underscores the importance of systematically collecting and reporting data to improve the recognition and management of hereditary thoracic aortic disease.

Although a positive family history remains one of the strongest indicators of hereditary thoracic aortic disease, more than one third of patients in our cohort underwent genetic testing despite the absence of a known family history and eighteen patients with disease-associated variants could be identified [2,3]. This finding emphasizes that the absence of a positive family history should not preclude consideration of HTAD.

As current guidelines recommend, apart from positive family history, early disease onset, aneurysms involving multiple vascular territories, and an aggressive clinical course should raise a suspicion of an underlying heritable aortopathy [1,10].

However, implementation of genetic testing remains dependent on clinical awareness and referral patterns [26]. As cardiothoracic surgeons are frequently the first physicians treating patients with aortic aneurysm or dissection, failure to recognize the possibility of an underlying hereditary aortopathy may result in missed diagnoses and an ongoing underestimation of HTAD prevalence.

Our results also demonstrate the clinical impact of identifying patients at risk beyond the index patient. Following genetic diagnosis of the index patient, 87 relatives underwent screening. Notably, a remarkable percentage underwent preventive aortic surgery after recognition of their increased risk. Therefore, the treating surgeon plays a key role not only in operative management but also in facilitating preventive care for family members.

A relevant finding of our study was the high proportion of patients carrying variants of uncertain significance (VUS), accounting for nearly one-fifth of the cohort. Interpretation of VUS remains one of the major challenges in contemporary cardiovascular genetics, as these findings currently do not allow definitive risk stratification or predictive testing. However, our cohort demonstrates that many patients with VUS presented with significant clinical manifestations, including familial clustering and adverse aortic events. Several individuals carrying VUS in HTAD-associated genes showed malignant disease courses, suggesting that systematic collection of clinical data and long-term follow-up of these patients are essential. A notable finding of our study was the high prevalence of variants of uncertain significance, particularly in *MYLK* and *MYH11*. The occurrence of aortic disease with aggressive clinical courses and familial clustering among some VUS carriers highlights the difficulty of interpreting rare variants in clinically enriched populations. These observations should be considered hypothesis generating and cannot establish pathogenicity without additional evidence, such as segregation, functional data, population data, or independent clinical observation [27,28]. Holmes et al. demonstrated that pathogenic *MYLK* variants are associated with an early disease onset and an aggressive clinical course, frequently presenting with acute aortic dissection at relatively small aortic diameters. Whether some currently classified variants of uncertain significance in *MYLK* may confer a similar, albeit less penetrant, phenotype remains unknown [29].

Consequently, systematic collection of genotype–phenotype correlations through multicentre registries and long-term follow-up will be essential to improve variant interpretation and optimize future management of affected families.

Despite the retrospective single-centre design and referral-based genetic testing strategy, our findings provide important real-world insights into the clinical consequences of identifying HTAD and add to the current literature [30]. Another limitation is that family screening relies on human individual decision-making. Despite standardized recommendations for genetic counselling and cascade screening, not all relatives choose to undergo genetic testing or aortic imaging.

Increased awareness among cardiothoracic surgeons, adherence to current guideline recommendations, and systematic collection of genetic and clinical data—also in patients with VUS—are crucial steps toward improving outcomes in hereditary aortic disease.

### Limitations

The main limitation of our study is the retrospective and monocentric design. While data on family history was 98% complete, genetic testing of the index patient was performed at the discretion of the treating cardiac surgeon, depending on whether the possibility of an underlying heritable aortic disease was considered. Information on genetic and imaging-based screening and adverse aortic events among relatives was collected retrospectively from index patients, limiting detailed follow-up data. Furthermore, due to the retrospective study design, the total number of patients with thoracic aortic disease presented to our department during the study period, as well as the number of patients who were evaluated for suspected heritable aortic disease but did not undergo genetic testing, could not be reliably determined. Consequently, the proportion of potentially eligible patients who underwent genetic testing cannot be assessed, representing an additional limitation and potential source of selection bias.

## 5. Conclusions

Cardiac surgeons should be aware of the importance of genetic testing in patients with aortic disease. Beyond the individual patient, the broader goal is to identify at-risk family members and prevent acute aortic syndromes. Data collection is essential to improve our understanding of variants of uncertain significance. Publications on this topic are essential to raise awareness of the role non-syndromic aortopathies and to highlight the importance of screening of at-risk family members.

## Figures and Tables

**Table 1:** Baseline characteristics, reason for consultation and pathogenic variants.

	N = 139
Female sex	46 (33)
Age (y)	45.8 ± 15.1
Reason for consultation	
Elective aneurysm repair	49 (35)
Type A aortic dissection	55 (40)
Surveillance	31 (22)
Type B aortic dissection	6 (4)
Pathogenic variants	33 (24)
Marfan syndrome	11 (8)
Loeys–Dietz syndrome	8 (6)
*SMAD3*	5 (4)
*TGFBR1*	1 (1)
*TGFBR2*	1 (1)
*TGFB2*	1 (1)
Vascular Ehlers–Dahnlos	4 (3)
*MYH 11*	7 (5)
*ACTA 2*	3 (2)

Values are presented as n (%) or mean ± SD; *ACTA2* = actin alpha 2, smooth muscle; *FBN1* = fibrillin-1; *MYH11* = myosin heavy chain 1.

**Table 2:** Indications for genetic testing (more than one possible) and family history.

	N = 139
Family history suggestive of HTAD	83 (60)
Dissection < 60 years of age	55 (40)
Root aneurysm < 60 years of age	30 (22)
Suspicious phenotype	23 (17)
Multilocular aneurysms	10 (7)
Rapid aortic growth/Extensive aneurysm (other reason absent)	7 (5)
Family history	
Proven	16 (12)
Likely	42 (30)
Possible	25 (18)

Values are presented as n (%); HTAD = hereditary thoracic aortic disease.

**Table 3:** Different variants of unclear significance and their clinical presentation.

Gene (+Disease Mechanism)	HGVS Variant	Clinical Presentation	Age (Sex)	Family Members with Aneurysms	Family Members Dissected/Died	ACMG Classification	ClinVar Classification	Population Frequency	Previously Published Variant
*MYLK* NM_053023.5 Loss of function;	c.1005_1007delinsTCT p.(Pro336Leu), het	Root aneurysm, operated, surgical correction of coarctation in childhood	41 (♂)	none	Grandfather sudden aortic death	VUS, Class 3	Benigne/likely benign	n/a	no
haploinsufficiency → impaired VSMC contractility	c.1672C>T; p. (ARG558Cys) rs1272395846; het	Root aneurysm, operated	35 (♂)	Brother same mutation and root aneurysm with tricuspid valve	-	VUS, Class 3	Uncertain significance/likely benign	0.00001	no
	c.3784A>G p.(Thr1262Ala) rs1553788596 het	Type A dissection	53 (♀)	Lost to screening, none known	Father sudden aortic death with 72y	VUS, Class 3	Likely benign/uncertain significance	0.00000	no
	c.2936C>T p.(Pro979Leu)rs368229473	Ascending ectasia & BAV (surveillance)	30 (♀)	none	none	VUS, Class 3	Uncertain significance/likely benign	0.000015–0.00004	no
	c.1133G>A p.(Arg378His)rs56378658 het	Root aneurysm (surveillance) Pectus excavatum	64 (♂)	1—brother operated (abdominal aorta)	1—carotid dissection (mother)	VUS, Class 3	Conflicting classifications of pathogenicity	0.0002	yes [12]
	c.2605G>T, p.(Val869Leu) het	Root aneurysm (surveillance)	42 (♀)	none	none	VUS, Class 3	Likely benign/uncertain significance	0.00008–0.00014	no
	c.3901C>T p.(Arg1301Cys) het rs 36821325	Type A Dissection	37 (♂)	none	none	VUS, Class 3	Uncertain significance/ conflicting classifications of pathogenicity	0.00005–0.00009	no
	c.3610C>T p. (Arg1204Trp) rs151294221 het	Root aneurysm (operated)	50 (♀)	none	2—twin brother carotid and vertebral artery dissection; father sudden death at 54	VUS, Class 3	Uncertain significance/conflicting classifications of pathogenicity	0.00011–0.00023	yes [13,14,15,16]
	c.257G>A p.(Arg86Gln) rs138265409	Root aneurysm + PFO (operated)	62 (♂)	none	2—male cousin died of aortic rupture; another female cousin sudden death at 55	VUS, Class 3	Conflicting classifications of pathogenicity/uncertain significance/likely benign	0.00008–0.00046	yes [17]
*MYH 11* NM_001040113.2 dominant-negative effect; impaired smooth-muscle myosin filament assembly/contractility	c.2914G>T, p.(ala972Ser), rs113696032 het & c.3322G>A, p.(Asp1108Asn), rs764532365, het (2 VUS)	Root aneurysm (operated)	62 (♂)	3—3 sisters, all operated	3—father and brother sudden death age 60; another brother dissected (dead)	VUS, Class 3 VUS, Class 3	Conflicting classifications of pathogenicity/uncertain significance/likely benign Uncertain significance	0.00027–0.00046 0.00000– 0.00002	no no
missense; non-coding transcript variant	c.2146C>T (rs567285465, p.Arg716Cys), het & *TGFB2* c.272G>A (rs10482721; p.Arg91His) het	Root aneurysm (operated)	35 (♂)	2—uncle with root aneurysm	2—father dissected (died); grandfather sudden death at young age	VUS, class 3 VUS, class 3	Uncertain significance Benign/likely benign/uncertain significance	0.00020 0.00080	no yes [18]
	c.2096C>T (p.A699V) het, rs148893135	Root aneurysm (operated)	39 (♂)	2—both sons with root aneurysms (1 operated at age 18, 1 surveillance)	1—grandfather sudden death	VUS, class 3	Conflicting classifications of pathogenicity/uncertain significance	0.00026–0.00031	yes [19]
	c.3225G>C p.(Gln1075His) het	Type A dissection (whole arch; survived)	51 (♂)	none	none	VUS, class 3	Uncertain significance	n/a	no
MFAP 5 NM_0034480 Loss of function extracellular matrix disruption	c.200C>T p.(Pro67Leu) het	Aortic dissection	43 (♂)	none	none	VUS, class 3	Not listed	n/a	yes [20]
FBN 1 NM_000138.4 dominant-negative effect and haploinsufficiency; impaired microfibril formation/ECM integrity	c.8071G>A p.(Gly2691Ser) het, rs145105768	Aortic dissection (survived), clipping of ACI aneurysm	54 (♀)	Neg. for aortic aneurysms, many family members with intracranial family members	none	VUS, class 3	Conflicting classifications of pathogenicity/benign/likely benign	0.00005–0.00015	yes [21,22]
	c.7412C>G (rs193922233), het	Root aneurysm (operated)	50 (♂)	1—mother operated	1—brother dissected and died	VUS, class 3	Conflicting classifications of pathogenicity/benign/likely benign	0.00005–0.00015	yes [21,22]
	c.6497A>T; p.Asp2166Val	Type A aortic dissection at age 37; Type B dissection at age 52	37 (♂)	none	1—father dissected and died	VUS, class 3	Not listed	n/a	no
*LOX*- impaired collagen and elastin cross-linking	c.6997+3G>Ars1270425478, rs1270425478 //*LOX* NM_002317.7 c.298G>A p.(Ala100Thr) rs762913437) //*MYLK* NM053025.4 c.2866G>A p.(Val956lle)	Root aneurysm under surveillance	74 (♂)	none	none	VUS, class 3 VUS, class 3 VUS, class 3	Uncertain significance Uncertain significance Uncertain significance	0.00001 n/a 0.00000	yes [23,24] no no
*COL3A1* NM000090.3	c.4048C>A (rs1414355898, p.Leu1350lle), het	Type A dissection	31 (♂)	4—mother and 3 siblings (surveillance)	none	VUS, class 3	Not listed	0.00009	no
presumed dominant-negative mechanism (structural alteration of type III collagen)	c.515A>C, p.(Tyr172Ser), rs771654029, heterozygot	Type B dissection (thoracoabdominal repair), then arch aneurysm (operated)	45 (♂)	none	none	VUS, class 3	Benign/likely benign/uncertain significance	0.00001–0.00011	yes [25]

ACI = arteria carotis interna; BAV = bicuspid aortic valve; *COL3A1* = collagen type III alpha 1 chain; *FBN1* = fibrillin-1; het = heterozygous; *LOX* = lysyl oxidase; HGVS = Human Genome Variation Society; MFAP5 = microfibril-associated protein 5; *MYH11* = myosin heavy chain 11; *MYLK* = myosin light chain kinase; PFO = patent foramen ovale; rs = Reference SNP cluster ID; *TGFB2* = transforming growth factor beta 2; VUS = variant of uncertain significance.

**Table 4:** Adverse disease-related events in family members after diagnosis of the index patient.

	Adverse Event	Genetic Testing Performed Pre-Event	Clinical Screening Performed Pre-Event	Diagnosis of Index Patient
1	Type B dissection & consecutive death (brother)	Yes—positive	Yes—Diameter not known	Vascular Ehlers–Dahnlos *COL3A1* c.313C>T (p.Gln105 *) het
2	Non-A–non-B dissection (mother; survived)	No—testing after dissection (positive)	N/a	Pathogenic *MYH11* variant c.3877_3880delins3880-84_3880-1 (p.Gln1293Profs*6), het
3	Death from intracranial bleeding (daughter)	Yes—positive	Yes—no vascular dilatation	Loeys–Dietz syndrome Type 3 *SMAD 3* c.728G>T (p.Arg243Leu) het, außerdem TTN c.87040C>T (p.Arg29014 *)
4	Sudden suspected aortic death at 55 * (cousin)	No	N/a	VUS *MYLK* (NM_053025.4) c.257G>A p.(Arg86Gln) rs138265409

*COL3A1* = collagen type III alpha 1 chain; *MYH11* = myosin heavy chain 11; *MYLK* = myosin light chain kinase; rs = Reference SNP cluster ID; *SMAD* 3= SMAD family member 3; VUS = variant of uncertain significance. * As neither the cause of death nor the presence of aortic disease or the genetic status of the deceased relative was known, this represents an anecdotal observation of uncertain significance.

## Data Availability

The data underlying this article will be shared on reasonable request to the corresponding author.
